# Pleiotropic Functions of High Fat Diet in the Etiology of Osteoarthritis

**DOI:** 10.1371/journal.pone.0162794

**Published:** 2016-09-09

**Authors:** Yoshinori Asou, Munetaka Iwata, Hiroki Ochi, Maierhaba Ailixiding, Zulipiya Aibibula, Jinying Piao, Guangwen Jin, Yasushi Hara, Atsushi Okawa

**Affiliations:** 1 Department of Orthopedics Surgery, Tokyo Medical and Dental University, 1-5-45 Yushima Bunkyo-ku, Tokyo, 113-8519, Japan; 2 Division of Veterinary Surgery, Nippon Veterinary and Life Science University, 1-7-1 Sakaiminamicho Musashino-shi, Tokyo, 180-8602, Japan; 3 Department of Physiology and Cell Biology, Tokyo Medical and Dental University, 1-5-45 Yushima Bunkyo-ku, Tokyo, 113-8519, Japan; 4 Department of Rehabilitation Medicine, Graduate School, Tokyo Medical and Dental University, 1-5-45 Yushima Bunkyo-ku, Tokyo, 113-8519, Japan; Universite de Lyon, FRANCE

## Abstract

Obesity is a risk factor for osteoarthritis (OA). To investigate the roles of increased mechanical loading in the onset of obesity-induced OA, knee joints were histologically analyzed after applying a tail suspension (TS) model to a high-fat diet (HFD)-induced OA model. Mice were divided into four groups: normal diet (ND) with normal loading (NL) group; HFD with NL group; ND with TS group; and HFD with TS group. Whole knee joints were evaluated by immunohistological analysis. The infrapatellar fat pad (IPFP) was excised and mRNA expression profiles were compared by qPCR analysis. After twelve weeks of the diet, body weight was increased by HFD in both the NL group and TS group. Upon histological analysis, the irregularity of the surface layer of articular cartilage was observed only in the NL+HFD group. Osteophyte area increased as a result of HFD in both the NL and TS groups, although osteophyte area in the TS+HFD group was smaller than that of the NL+HFD group. In the evaluation of the IPFP by qPCR, adipokines and inflammatory cytokines also increased as a result of HFD. While TGF-β increased as a result of HFD, the trend was slightly lower in the TS group, in parallel with osteophyte area. To detect apoptosis of articular chondrocytes, TUNEL staining was employed. TUNEL-positive cells were abundantly observed in the articular cartilage in the HFD mice regardless of mechanical loading. IPFP inflammation, enhanced chondrocyte apoptosis, and osteophyte formation were seen even in the TS group as a result of a HFD. In all, these data demonstrate that HFD contributed to osteophyte formation through mechanical loading dependent and independent mechanisms.

## Introduction

Osteoarthritis (OA), a chronic degenerative joint disorder characterized by articular cartilage destruction and osteophyte formation, is a major cause of disability. Obesity and high body mass index are associated with a higher risk of OA [1–4].Obesity introduces increased weight-bearing on the knee joints [5]. While mechanical factors are implicated in the cause of OA, trauma, joint instability, and developmental dysplasias are all recognized as predisposing factors and have been affirmed in animal models [6]. As these factors alter the extent of mechanical loading to the joints, OA is suggested to be induced by an increase in mechanical loading.

Tail suspension is an animal model of hindlimb unloading. This model is employed to investigate the biological mechanisms involved in skeletal tissue homeostasis during unloading circumstances, such as space flight and bed rest [7]. The unloading of the hindlimb of C57BL/6 J mice promotes bone resorption, and as a result, the suspended hindlimbs exhibit osteopenia [7]. Skeletal unloading of F344/N rats increases alkaline phosphatase activity at the deep zone in association with a decrease in proteoglycan content in the articular cartilage [8].

Several cohort studies have demonstrated that being overweight is an independent risk factor for hand OA [9, 10]. Since mechanical stress cannot explain such a correlation, the influence of one or several systemic factors has been proposed. In addition to the association with obesity and the risk of OA, obesity is also associated with an increased amount of adipose tissue, which expresses and secretes a large number of adipokines in response to metabolic changes [11].

Various laboratories have established in vivo OA models in order to study the mechanisms of OA development [6, 12–15] [16–18], providing a HFD has been shown to increase the incidence of OA in male mice of C57Bl6 strain [16, 17]. We previously showed that the infrapatellar fat pad (IPFP) plays a pivotal role in the formation of osteophytes and functions as a secretory organ using a murine HFD-induced OA model [19]. The initiation of OA changes, such as osteophyte formation and articular chondrocyte apoptosis, occurs within three months of HFD with the adipocyte hypertrophy and increased angiogenesis of the IPFP [19]. The expression of adipokines and adipocyte hypertrophy markers are correlated with the expression of TGF-β and inflammatory cytokines in the IPFP [19], suggesting that adipocyte hypertrophy is closely linked to osteophyte formation through secretion of inflammatory cytokines. The IPFP is a unique fat depot that is located between the joint capsule and the synovial tissues, and is in close contact with articular cartilage. Recently, the endocrine function of the IPFP has been implicated in the initiation and progression of OA [20–22]. However, it is still unclear whether the events observed in the IPFP and articular cartilage are directly induced by HFD or are an indirect response to the destruction of articular cartilage in OA. For instance, it is not clear whether osteophyte growth and increased chondrocyte apoptosis in HFD mice are caused by mechanical overload and/or altered IPFP metabolism. Furthermore, it is not clear whether mechanical overload triggers the IPFP inflammation or if HFD stimulates inflammatory responses in the IPFP regardless of the loading. To address these issues, we applied a tail suspension (TS) model to a HFD-induced OA model to exclude the effect of mechanical overload by HFD, and investigated the roles of IPFP inflammation and mechanical loading in the onset of obesity-induced OA.

## Materials and Methods

All animal experiments were approved by the Animal Care and Use Committee of Tokyo Medical and Dental University and were carried out in accordance with the approval guidelines. C57Bl6J mice were fed a diet containing 32% fat for the HFD group or 4.8% fat for the control group (HFD32 and CE-2; CLEA Japan, Inc. Tokyo, Japan) [23] from the age of seven weeks. Mice fed a high fat diet and a normal (control) diet were divided into two groups (8 weeks and 12 weeks) based on the duration of the treatment. All animals were housed individually, allowed unrestricted activity and were provided food and water *ad libitum*. None of the mice died during the experimental period.

### Tail suspension model

Mice were divided into four groups: normal loading (NL) with normal diet (ND), NL with HFD, tail suspension (TS) with ND and TS with HFD. The tail suspension model was carried out as previously reported [7]. Briefly, a tape was applied to the surface of the tail to set a metal clip. The end of the clip was fixed to an overhead bar and the height of the bar was adjusted to maintain the mice at ∼30° head down tilt with the hind limbs elevated above the floor of the cage. Mice in the TS+HFD group were subjected to tail suspension and HFD from 7 weeks of age. Mice in the TS+ND group and TS+HFD were housed individually under the same condition.

### Assessment of OA severity

Mice were sacrificed at 8 weeks or 12 weeks after starting the diet (10 mice/group at each time point). Whole knee joints were removed by dissection, fixed in 4% paraformaldehyde, and decalcified in EDTA. After dehydration and paraffin embedding, serial 5-μm-thick sagittal sections were made from the whole medial compartment of the joint, as reported previously [19].

The sections were stained with Safranin O–fast green or HE. OA severity in the tibial plateau was evaluated according to a cartilage destruction score. Quantitative osteophyte determination was made using Image-Pro Plus 4.1 software (Media Cybernetics, Carlsbad, CA). The protruded region, which stained green by Safranin-O staining, was defined as bony osteophyte and quantified as reported previously [19]. For the evaluation of OA severity, representative sections were selected from the medial tibial plateau and medial femoral plateau, and scored with osteoarthritis research society international (OARSI) scoring [24]. Two representative sections from each mouse were blindly evaluated by three different readers.

### Micro-computed tomography (CT) analysis

CT scanning was performed with a ScanXmate-A090S Scanner (Comscantecno, Co., Ltd., Kanagawa, Japan). Three-dimensional microstructural image data were reconstructed and structural indices were calculated using TRI/3D-BON software (RATOC System Engineering, Kyoto, Japan). Bone morphometric analyses were performed at a region 0.3 to 0.5 mm above the distal growth plates of the femora. Picutures were acquired in vitro at 59kV in tube voltage, 62μA in tube current and 3.7W in tube electric energy.

### RNA extraction and real-time RT-PCR

The IPFP tissue was excised using a surgical microscope and microsurgical technique at sacrifice as reported elsewhere [19]. Total RNA isolated using TRIzol reagent (Invitrogen) was quantified by spectrophotometric readings at 260/280 nm. Total RNA (1 μg) was reverse-transcribed (Super Script VILO cDNA Synthesis Kit; Invitrogen) and used for determining the expression of Nicotinamide phosphoribosyltransferase (*Nampt)*, *Leptin*, Vascular endothelial growth factor (*Vegf)*, Tumor necrosis factor alpha (*TNF-alpha)*, Interleukin-6 *(Il-6)*, and Transforming growth factor beta 1 (*TGF-beta1)*. Mouse-specific primers (Sigma-Aldrich) were designed using the Primer Express software, version 3.0 (Applied Biosystems). Polymerase chain reaction (PCR) was performed on a Stratagene Mx3000p System (Agilent Technologies Japan, Ltd.) by using the Kapa Sybr Fast qPCR Kit (Kapa Biosystems, Inc.; Boston, MA, USA). The expression of mRNAs was normalized to that of *β-actin*, and fold differences were calculated using the ΔΔCt method.

### TUNEL assay

The TUNEL assay was performed using a TUNEL detection kit according to the manufacturer’s instructions (Takara Shuzo, Kyoto, Japan) and the previous report [19]. Briefly, two representative sections were picked from each knee sample, and the number of TUNEL positive cells was averaged between them.

### Statistical analysis

Data are expressed as means ± SD. Statistical analysis was performed with non-parametric Steel's many-one rank test. P values less than 0.05 were considered significant.

## Results

### Unloading and HFD coordinately reduced lower limb bone volume

**Fig 1:**
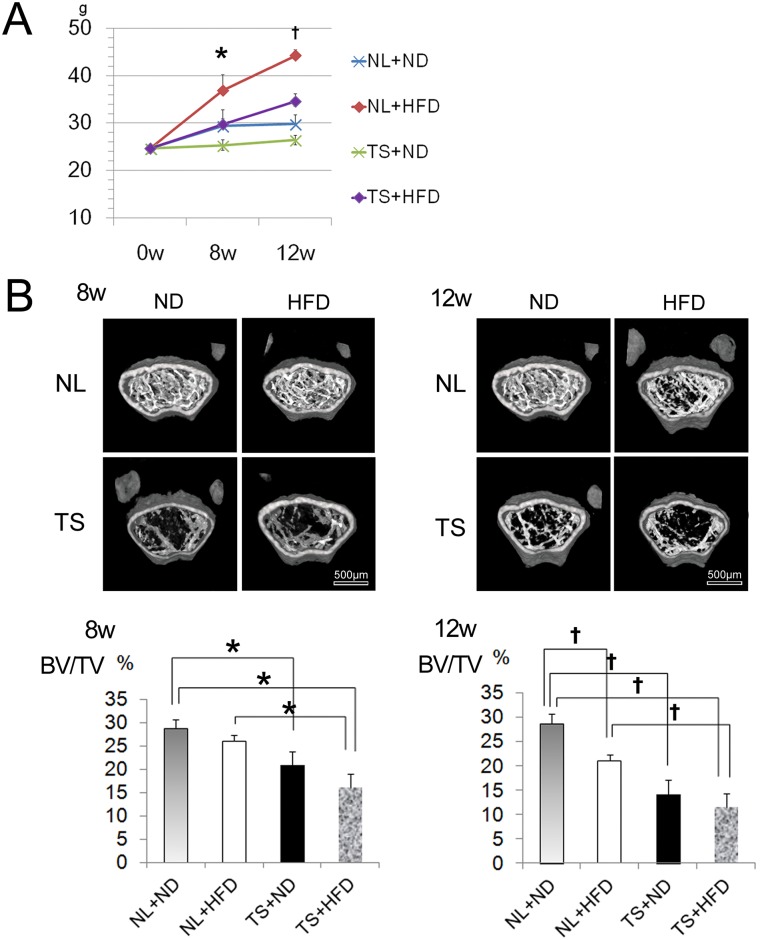
Body weight and μCT analysis. A, Body weights of NL+ND, NL+HFD, TS+ND, and TS+HFD mice at the indicated lengths of the diets. B, Three-dimensional micro-CT analysis and BV/TV of the femoral bones from the mice of indicated diets and loadings. NL, normal loading; ND, normal diet; HFD, high-fat diet; TS, tail suspension. *,† = P < 0.05 using non-parametric Steel's many-one rank test.

To clarify the role of mechanical stress in the histological alterations of the knee joints in HFD mice, the TS model was applied to ND and HFD mice. Under normal loading, HFD mice weighed 25% more than ND mice by 8 weeks and 48% more by 12 weeks (Fig 1A, p <0.05). TS+HFD mice weighed 18% more by 8 weeks and 31% more by 12 weeks when compared with TS+ND mice, although they weighed 20% less by 8 weeks and 18% less by 12 weeks when compared with NL+HFD mice.

As previously reported (22–24), femoral cancellous bone was decreased as a result of HFD and TS (Fig 1B). Bone volume to total volume (BV/TV), which decreased as a result of TS, decreased further with the addition of HFD (Fig 1B).

### The effects of the loading and the diets on the histological features of the articular cartilage

**Fig 2:**
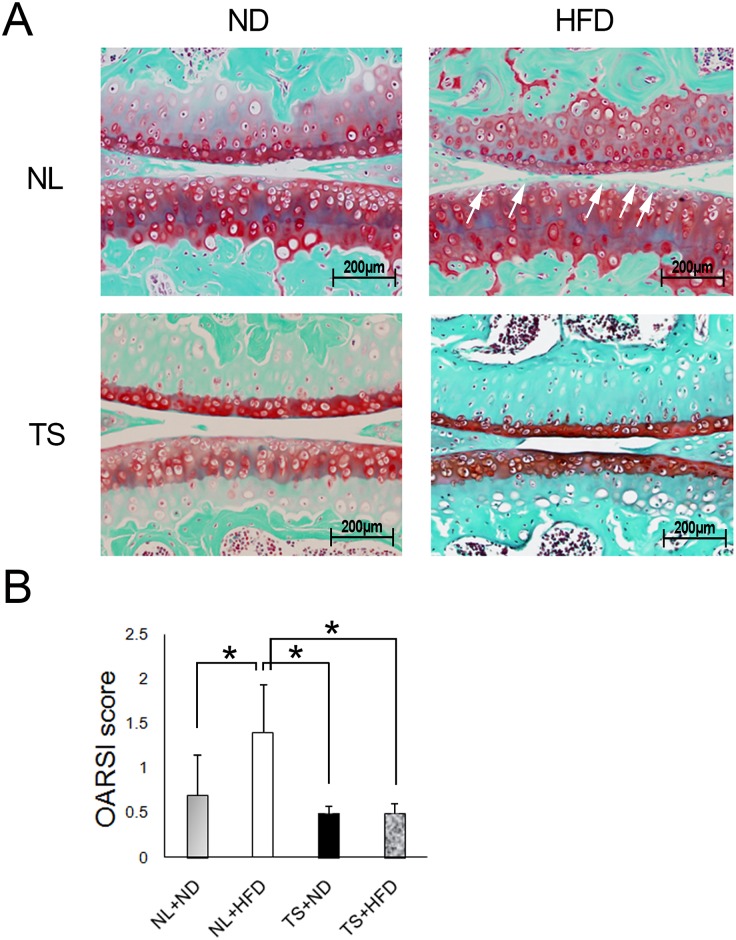
Histological analysis of the knee joints of mice fed HFD and normal diet with the indicated loadings. A, Sections of articular cartilage from the mice at 12 weeks after the onset of the diets stained with Safranin O to detect proteoglycan expression. The arrows indicate aberrant stainability at the surface of articular cartilage in the NL+HFD group. B, OA severity of medial tibial plateau based on OARSI scoring for mice. * = P < 0.05 using non-parametric Steel's many-one rank test.

A histological analysis was conducted next (Fig 2). The stainability of the surface layer of articular cartilage with Safranin O decreased as a result of HFD in NL+HFD, while the stainability of deep layers decreased as a result of TS both in HFD and ND mice (Fig 2A). In HFD groups, fibrillation of the joint cartilage surface was observed in the NL+HFD group, but not in the TS+HFD group. The OA severity based on OARSI score indicated NL+HFD mice scored significantly higher compared to any other groups (Fig 2B). This result reflected an increase in surface irregularity and a decrease in proteoglycans at the surface of the articular cartilage in NL+HFD mice.

### The effects of the loading and the diets on osteophyte formation

**Fig 3:**
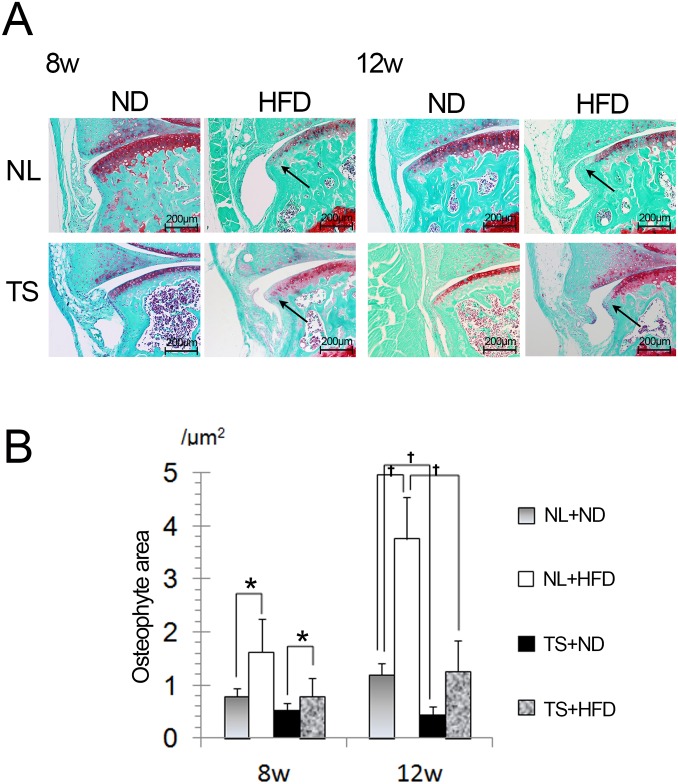
High magnification of the anterior edge of the tibial plateau. A, Representative Safranin O stained sections from mice fed indicated diets and loadings at 8 weeks and 12 weeks. Ossified osteophytes grew from 8 weeks of HFD (arrows). B, Mean osteophyte volume in indicated diets and loadings for 8 weeks or 12 weeks after the onset of the diet. NL, normal loading; ND, normal diet; HFD, high-fat diet; TS, tail suspension. Values represent the mean and SD. *,† = P < 0.05 using non-parametric Steel's many-one rank test.

Osteophyte formation is among the characteristic features of OA. In NL groups, HFD was associated with enhanced osteophyte formation in the NL+HFD group compared to the NL+ND group at the anterior edge of the sagittal section of the tibial plateau from eight weeks of the diet (Fig 3A and 3B). In TS groups, osteophyte area was also increased in the TS+HFD group compared to the TS+ND group after 12 weeks of the diet. Notably, in HFD groups, osteophyte area in the TS+HFD group decreased compared to the NL+HFD group. In ND groups, osteophyte area was also decreased in the TS+ND group when compared to the NL+ND group (Fig 3A and 3B). These results suggest that HFD contributed to osteophyte formation in both a mechanical loading dependent and independent manner.

### Chondrocyte apoptosis was increased by HFD independent of mechanical loading

**Fig 4:**
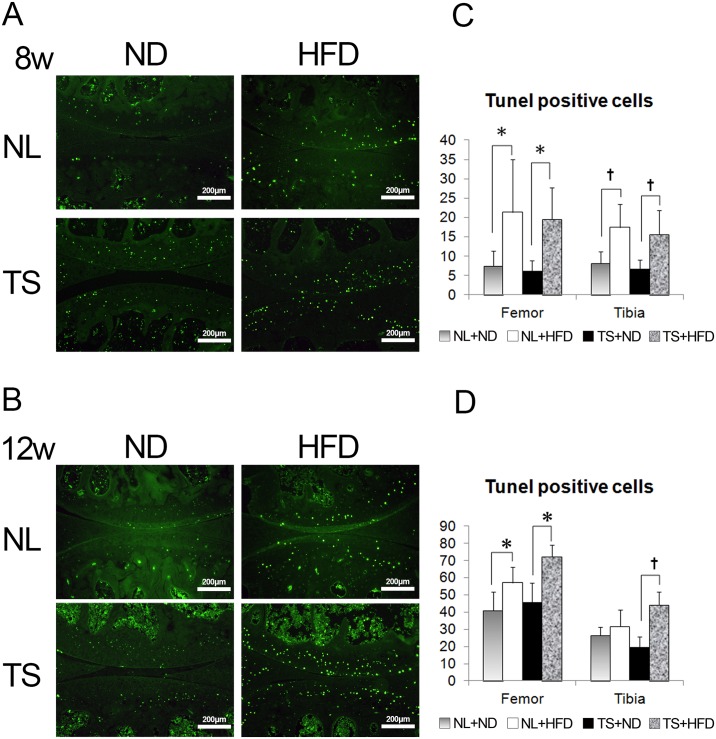
Analysis of apoptosis in TUNEL-stained sections of the knee joint. (A, B) Representative TUNEL-stained sections from mice fed the indicated diets and loadings for 8 weeks (A) and 12 weeks (B). The number of TUNEL-positive cells per section of superficial layer of artificial cartilage was determined by fluorescence microscopy (C, D). The number of TUNEL-positive cells increased in the knee joint cartilage from HFD mice both at 8 (C) and 12 weeks (D). Values are the mean and SD of ten mice per group. NL, normal loading; ND, normal diet; HFD, high-fat diet; TS, tail suspension. *,† = P < 0.05 using non-parametric Steel's many-one rank test.

Chondrocyte apoptosis is increased in OA cartilage and is anatomically linked to proteoglycan depletion [25] [26]. These observations prompted us to investigate the effect of the HFD on chondrocyte apoptosis (Fig 4). TUNEL staining was performed at week eight and twelve of the diet. TUNEL-positive cells were abundantly observed in the superficial layer of the articular cartilage (Fig 4A and 4B) in the HFD mice regardless of loading. TS itself had no effect on apoptosis of the articular chondrocytes. The number of TUNEL-positive cells in the articular cartilage was significantly increased in the HFD group (Fig 4C and 4D). These results showed that chondrocyte apoptosis was induced by HFD independent of mechanical loading.

### mRNA profiles in the IPFP

**Fig 5:**
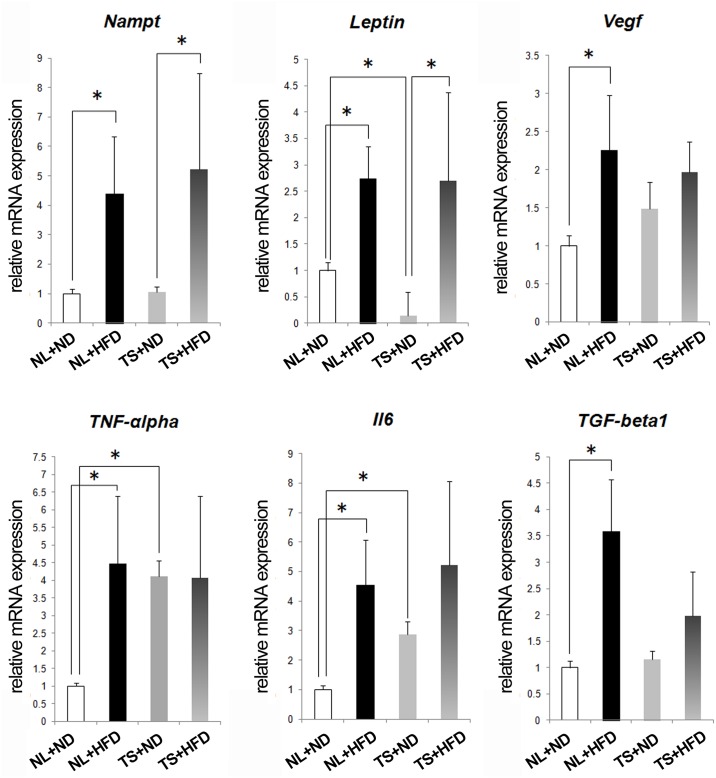
Real-time RT-PCR analysis of IPFP. The mRNA expression of Nampt, leptin, VEGF, TNF-α, IL-6 and TGF-β1in the IPFP was evaluated by real-time RT-PCR analysis in the indicated mice. Values are the means ± 1 SD of ten mice per group. NL, normal loading; ND, normal diet; HFD, high-fat diet; TS, tail suspension. * = P < 0.05 using non-parametric Steel's many-one rank test.

The expression levels of inflammatory cytokines in the IPFP were evaluated, since the IPFP has recently been implicated in the pathology of osteoarthritis [27, 28]. The IPFP was excised using a surgical microscope and microsurgical technique at 12 weeks after starting the diet (Fig 5). Both Nampt and leptin increased as a result of HFD regardless of loading. Expressions of TNF-α, VEGF, and IL-6 similarly increased as a result of HFD. TNF-α and IL-6 were also highly expressed in TS+ND mice compared to NL+ND mice. While TGF-β expression was increased in HFD groups when compared to ND groups, its expression was lower in TS+HFD mice compared to NL+HFD mice.

## Discussion

**Fig 6:**
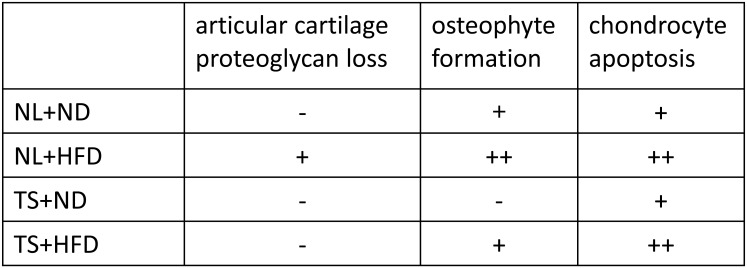
Summary of histological findings at 12 weeks of the diets. NL, normal loading; ND, normal diet; HFD, high-fat diet; TS, tail suspension.

In this study, it was found that HFD induced OA changes, including proteoglycan loss, chondrocyte apoptosis, and osteophyte formation, in association with IPFP inflammation by 3 months of exposure to high fat diet in both a normal loading and tail suspension mouse model. The histological findings of NL+ND, NL+HFD, TS+ND and TS+HFD are summarized in Fig 6.

Reduced Safranin O stainability of the cartilage surface occurred in the NL+HFD group, but not in the TS+HFD group. This was considered the result of a large contribution of mechanical loading. Superficial zone protein (SZP) has been demonstrated to contribute to the boundary lubrication in synovial joints. The expression levels of the SZP of articular cartilage are enhanced by optimal mechanical stimuli, but decreased by excessive loading partly through the signaling of TGF-β1 and IL-1β [29]. The increase in obesity-related mechanical loading on the surface of articular cartilage in the present study may have contributed to a decrease in SZP. The stainability of deep layers decreased in TS groups regardless of the diets. These findings were not observed in NL+ND nor NL+HFD groups. This is consistent with a report stating that skeletal unloading increased ALP activity at the deep zone and temporarily accelerated tidemark advancement associated with a decrease in proteoglycan content [8].

Osteophyte formation was evaluated at the anterior edge of the tibial plateau. Both in ND groups and HFD groups, osteophyte area in the TS group decreased compared to the NL group. These results suggest that mechanical loading plays a role in osteophyte growth. On the other hand, HFD was associated with enhanced osteophyte formation in both NL groups and TS groups. These findings suggested HFD also contributed to osteophyte formation through loading-independent mechanisms. There is significant overlap in the location of TGF-β-induced and experimental OA-induced osteophyte formation [30]. These observations confirm that TGF-β plays a role in osteophyte development during experimental OA [31]. To date, we have demonstrated that expression of TGF-β is enhanced in the IPFP as a result of HFD [19]. We also revealed TGF-β mRNA expression is positively correlated with Leptin expression in the IPFP [19]. Leptin stimulates chondrocyte synthesis of TGF-β in animal experiments [32]. Thus, Leptin may act as a trigger to stimulate TGF-β expression from the IPFP. We previously displayed Nampt is densely expressed around osteophytes [19]. Here, we disclosed Nampt expression in the IPFP was increased by HFD. Nampt is also expressed at high levels during osteogenic differentiation of the multipotent mouse fibroblast cell line C3H10T1/2 and the pre-osteoblast cell line MC3T3-E1, and influences osteogenic differentiation of these cells via Sirt1 activation [33]. These findings suggest Nampt may play a role in osteophyte formation, as well as TGF-β.

We have disclosed that chondrocyte apoptosis was induced by HFD independent of mechanical loading. Among the adipokines and cytokines evaluated, the expression pattern of Nampt correlated with the pattern of the apoptotic cells among the groups. Nampt expression was induced by HFD independent of mechanical loading. Nampt production is increased by IL-1β in chondrocytes [34], and triggers the synthesis and release of MMP-3, MMP-13, ADAMTS-4, and ADAMTS-5 by chondrocytes [34]. Recently, NAMPT was shown to play an essential catabolic role in OA pathogenesis downstream of HIF-2α [35]. Nampt enhances MT2 expression and then stimulates chondrocyte apoptosis [36]. Thus, upregulated Nampt may augment chondrocyte apoptosis in NL+HFD and TS+HFD mice.

We observed that TNF-α and IL-6 in the IPFP were highly expressed in not only TS+HFD but also TS+ND mice. The expression of TNF-α and IL-6 is enhanced in unloaded muscle [37, 38] and bone [39]. These findings suggest unloaded synovial tissues or adipocytes express IL-6 and TNF-α through a mechanical stress sensing pathway such as the MyD88 signal [38]. The molecular mechanisms underlying this phenomenon have yet to be elucidated.

In conclusion, the present study showed that IPFP metabolism and mechanical loading associated with weight gain each serve a unique function in the pathology of knee OA induced by HFD. This provides an opportunity to investigate articular cartilage responses to metabolic stress and the mechanisms involved in the progression of OA.
